# Simulated atmospheric nitrogen deposition inhibited the leaf litter decomposition of *Cinnamomum migao* H. W. Li in Southwest China

**DOI:** 10.1038/s41598-021-81458-3

**Published:** 2021-01-18

**Authors:** Xiao-Long Huang, Jing-Zhong Chen, Deng Wang, Ming-Ming Deng, Meng-Yao Wu, Bing-Li Tong, Ji-Ming Liu

**Affiliations:** 1 Department of Ecology, College of Forestry, Guizhou University, Guiyang, 550025 China; 2 Forest Ecology Research Center of Guizhou University, Guiyang, 550025 China

**Keywords:** Forest ecology, Forestry

## Abstract

Atmospheric nitrogen (N) deposition could affect various ecological processes in forest ecosystems, including plant litter decomposition and nutrient cycling. However, the mechanism of underlying litter decomposition and nutrient cycling of *Cinnamomum migao* under N deposition remains unclear. Therefore, we conducted a simulated N deposition experiment including four onsite treatments to assess the effects of N input on *C. migao* leaf litter decomposition, nutrient release, and soil enzyme activity. The results showed that simulated N deposition significantly increased the amount of total residual mass and lignin and cellulose, decreased the decomposition rate, and suppressed net nutrient release. N input increased C, N, and P ratios as decomposition progressed, and the proportion of mass remaining was positively correlated with the proportions of lignin and cellulose remaining at the later stage of decomposition. The differences in soil enzyme activity were primarily due to enzyme type and sampling time. We conclude that simulated N deposition significantly suppressed the leaf litter decomposition of *C. migao* by mainly altering the chemical properties and suppressing the decomposition of the organic matter in leaf litter. Lignin might have played an important role in the loss of leaf litter biomass at the later stage of decomposition.

## Introduction

Burning of fossil fuels, large-scale production and application of nitrogen (N) fertilizers, and the development of animal husbandry in recent decades have rapidly increased the rate of N deposition^1,2^. Atmospheric N deposition in China increased from 13.2 kg·ha^−1^·year^−1^ in 1980 to 21.1 kg·ha^−1^·year^−1^ in 2000^3^, whereas N deposition on land in South China was 50 kg·ha^−1^·year^−1^ in 2008^4^; this significant increase in N deposition in Southwest China has affected the structure and function of forest ecosystems. Leaf litter forms a link between soil and vegetation^5–7^, and its decomposition is an important process that maintains ecosystem productivity and soil fertility^8–10^. In addition, it plays a key role in maintaining the global carbon (C) and nitrogen (N) balance^11,12^. Previous studies have reported that litter decomposition is influenced by biotic factors and abiotic factors^13–16^. However, the increasing N input in terrestrial ecosystems caused by atmospheric N deposition is expected to alter litter decomposition and ultimately influence the C storage and soil nutrient status of the ecosystem^17^.

The effects of N deposition on litter decomposition are variable depending on the duration of N deposited, and the litter type^18^. Therefore, the reported responses of litter decomposition and nutrient release to N deposition are conflicting^8^ and include enhancement^19–21^, suppression^22^, and no response^23,24^. Litter stoichiometry traits are important regulators of litter decomposition^25^ that are affected by initial C, N, and phosphorus (P) contents as well as by their C/N and C/P ratios^26^. Generally, the higher C/N and C/P ratios of the litter can negatively affect the mineralization of N and P during litter decomposition^27^. N deposition can also result in changes in substrate quality and alter the chemical composition of litter, thereby affecting its decomposition^28,29^. Regarding higher C/N ratio litter, N input at the initial stage of decomposition can increase the N concentration of litter; this in turn promotes decomposition by reducing the C/N ratio^30–32^. However, at the later stage of decomposition, it is primarily determined by the content of refractory macromolecular compounds (such as lignin and cellulose) and microbial community^33–35^. N input may have a negative influence on organic matter decomposition and effect litter decomposition by producing substantial amounts of residue^34^.

N deposition can also affect the soil microenvironment, soil N availability, and soil enzyme activity, which result in the changes in a series of ecological processes, including mineralization and nutrient fixation and decomposition by microorganisms; this indirectly affects litter decomposition^35,36^. Soil enzymes are directly involved in C, N, and P cycles in the soil ecosystem. Invertase, urease and acid phosphatase are responsible for the mineralization of C, N and P during litter decomposition, whereas peroxidase is mainly involved in lignin degradation, carbon mineralization, and defense^37,38^; however, N deposition can change soil enzyme activity by affecting soil nutrients availability, thereby interfering with the decomposition of organic matter by soil enzymes^39^. Generally, N input increases the available N content in soil and reduces the activities of enzymes related to microbial N acquisition, such as of urease and protease; however, it increases the activity of enzymes related to microbial C (e.g., peroxidase and cellulase) and P (e.g., acid phosphatase) acquisition^40,41^. However, in recent studies of Chinese forests, the effect of simulated N input on soil enzyme activity was not completely according to this theory^42,43^. Although some studies have evaluated the effects of elemental stoichiometry on litter decomposition in forest ecosystems, the relationship between litter decomposition and elemental stoichiometry in the forests of Southwest China remains poorly studied; this provided the impetus for our experiment.

*Cinnamomum migao* H. W. Li, an evergreen tree belonging to the Lauraceae family, is mainly distributed in Southwest China. In Guizhou Province, it is known for its medicinal properties. Recent studies have primarily focused on analyzing its chemical composition and the physiological and biochemical characteristics of its seedlings^44^. However, to the best of our knowledge, as the representative and predominant large tree species in the plant community of the distribution area, no study has examined the impact of N deposition on *C*. *migao* litter. Further, the response of nutrient cycling to N deposition in *C*. *migao* litter remains unclear. In this study, we selected *C*. *migao* as the research target and conducted simulated N input field experiments based on the typical N deposition levels in Southwest China^4^. The objectives of this study were to assess the changes in the decomposition rate and nutrient release from litter leaves; evaluate the response of soil enzyme activity to N deposition; and further elucidate the roles of litter quality, litter decomposition, and soil enzymes. Accordingly, we proposed the following hypotheses: (1) N input restricts the litter decomposition rate and suppresses the release and decomposition of C, N, P, lignin, and cellulose; (2) simulated N deposition changes litter chemical characteristics to influence leaf litter decomposition; and (3) N input changes soil enzyme activity to indirectly restrict litter decomposition. This study provides the basis for research on the effects of N deposition on soil nutrient cycling in subtropical medicinal plants and a reference for further studies on forest soil enzymes as well as on the scientific management of forest litter and soils.

## Results

### Mass remaining and leaf litter decomposition rate constant

**Figure 1:**
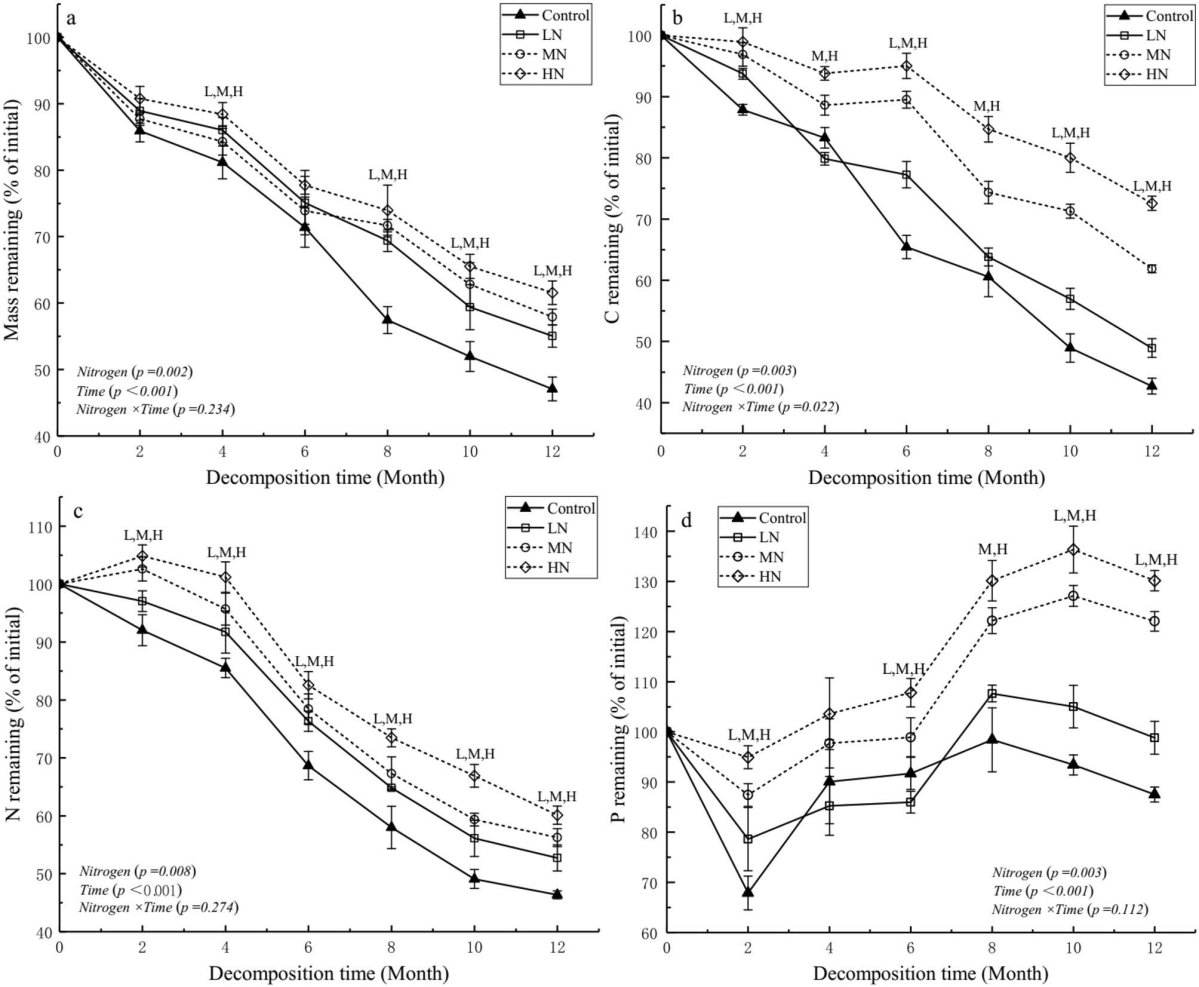
Effects of simulated N deposition on the dynamics of mass, carbon, nitrogen, phosphorus remaining in decomposing leaf litter. Values are expressed as mean ± standard error (SE) (n = 3). LN, MN, and HN treatments at each decomposition time indicated that the difference between N treatments and control treatment is significant (*P* < 0.05).

The proportion of mass remaining in *C*. *migao* leaf litter gradually decreased as the decomposition time increased. After 1 year of N deposition treatments, the proportions of mass remaining in low nitrogen (LN), medium nitrogen (MN), and high nitrogen (HN) treatments were 16.93%, 22.98%, and 30.73% higher, respectively, than that in the control treatment (Table 1). Treatments with N deposition significantly increased the proportion of mass remaining in *C*. *migao* leaf litter and significantly suppressed litter decomposition (*P* < 0.05). The suppression effects increased with increasing N deposition throughout the experiment year (Fig. 1a).

**Table 1:** Chemical properties of leaf litter in different N deposition treatments after 1 year of decomposition.

Treatment	Mass remaining (% of initial)	C remaining (% of initial)	N remaining (% of initial)	P remaining (% of initial)	Lignin remaining (% of initial)	Cellulose remaining (% of initial)	C/N ratio	C/P ratio
Control	47.08 ± 1.80 c	42.70 ± 1.29 d	46.38 ± 0.68 c	87.52 ± 1.49 d	94.94 ± 5.00 c	46.06 ± 2.54 c	26.82 ± 0.90 b	192.55 ± 7.78 b
LN	55.05 ± 1.71b	48.92 ± 1.51 c	52.74 ± 2.25 b	98.80 ± 3.27 c	117.59 ± 4.11 b	57.79 ± 2.54 b	27.14 ± 1.67 b	195.68 ± 9.09 ab
MN	57.90 ± 1.90 ab	61.86 ± 0.65 b	56.25 ± 1.55 ab	122.04 ± 1.95 b	125.97 ± 3.12 ab	63.29 ± 1.73 b	32.06 ± 0.79 a	200.00 ± 5.20 ab
HN	61.55 ± 1.75 a	72.56 ± 1.17 a	60.11 ± 1.57 a	130.14 ± 2.00 a	136.36 ± 4.29 a	75.21 ± 1.76 a	35.17 ± 0.79 a	220.02 ± 6.93 a

Values are expressed as mean ± SE (n = 3). Values marked with different letters are significant (*P* < 0.05). *LN* simulated low nitrogen deposition; *MN* simulated medium nitrogen deposition; and *HN* simulated high nitrogen deposition.

After 1 year of decomposition under each N treatment (Table 2), the sequence of *k*-values was as follows: control (0.7796) > LN (0.6106) > MN (0.5218) > HN (0.4937). The increased N deposition levels decreased the *k-*value of *C*. *migao* leaf litter. The time required for 50% (T_50%_) and 95% (T_95%_) in leaf litter decomposition was 0.89 year^−1^ and 3.85 year^−1^, respectively, in the control treatment group; N deposition treatments increased from 0.25–0.51 year and 1.06–2.23 year (Table 2). N treatments decreased the annual *k-*value of leaf litter, increased T_50%_ and T_95%_, and negatively affected leaf litter decomposition.

**Table 2:** Decomposition rate constant (*k*), coefficients of determination (*R*^2^), and time to 50% (T_50%_) and 95% (T_95%_) decomposition of leaf litter under different nitrogen deposition treatments.

Treatment	Regression equation	*k*	*R*^2^	T_50%_ (year^−1^)	T_95%_ (year^−1^)
Control	y = 100.95e^−0.78t^	0.7796	0.9423	0.89	3.85
LN	y = 101.43e^−0.618t^	0.6106	0.9016	1.14	4.91
MN	y = 97.99e^−0.5218t^	0.5218	0.9279	1.33	5.78
HN	y = 100.65e^−0.49t^	0.4937	0.9174	1.40	6.08

*LN* simulated low nitrogen deposition; *MN* simulated medium nitrogen deposition; and *HN* simulated high nitrogen deposition.

### Dynamics of C, N, and P remaining during leaf litter decomposition

After 1 year of the leaf litter decomposition of *C*. *migao*, C release in all treatments was via directed release as the decomposition time increased (Fig. 1b). The proportion of C remaining in the LN, MN, and HN treatments was 48.92% ± 1.51%, 61.86% ± 0.65%, and 72.56% ± 1.17%, respectively; the proportion of C remaining in the MN and HN treatments was significantly higher than that in the control treatment (42.70% ± 1.29%; *P* < 0.05; Table 1). Simulated N deposition suppressed C release, and the suppression effect increased as N deposition increased. N release during the entire leaf decomposition process was generally via direct release (Fig. 1c). The proportion of N remaining in the LN, MN, and HN treatments was 52.74% ± 2.25%, 56.25% ± 1.55%, and 60.11% ± 1.57%, respectively, and was significantly higher than the control (46.38% ± 0.68%; *P* < 0.05; Table 1), indicating that N release was suppressed by N treatment and that suppression increased as N deposition increased. In contrast, compared with the release pattern of C and N, P decreased in the initial 2 months. Furthermore, the proportion of P remaining in each treatment was obviously enriched, MN and HN treatments continued to the 10th month of decomposition, and the P remaining in each treatment was subsequently released (Fig. 1d). The proportion of P remaining in LN, MN, and HN treatments was 98.80% ± 3.27%, 122.04% ± 1.95%, and 130.14% ± 2.00%, respectively, and were significantly higher than those in the control treatment (87.52% ± 1.49%; *P* < 0.05; Table 1). Taken together, these results indicate that simulated N deposition promotes P accumulation.

### Dynamics of lignin and cellulose remaining during leaf litter decomposition

**Figure 2:**
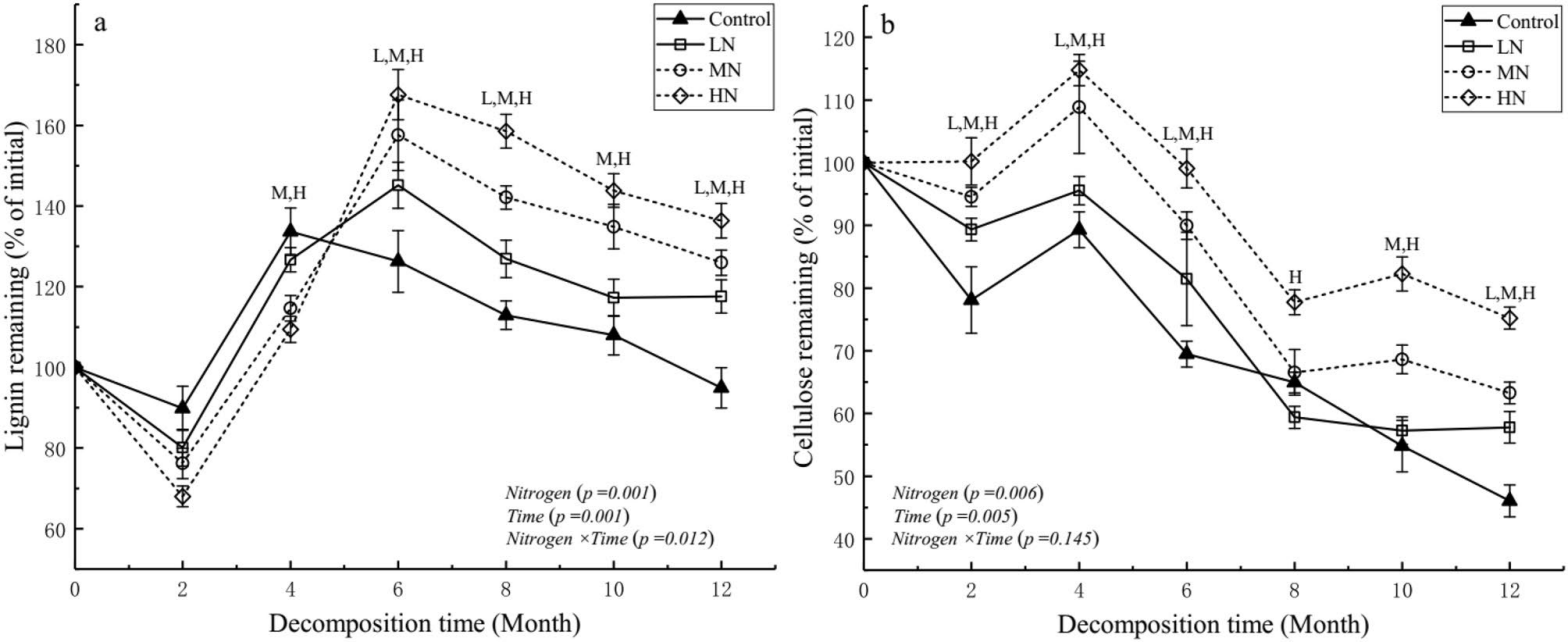
Effects of simulated N deposition on the dynamics of lignin and cellulose remaining in decomposing leaf litter. Values are expressed as mean ± standard error (SE) (n = 3). LN, MN, and HN at each decomposition time indicated that the difference between N treatments and control treatment is significant (*P* < 0.05).

**Figure 3:**
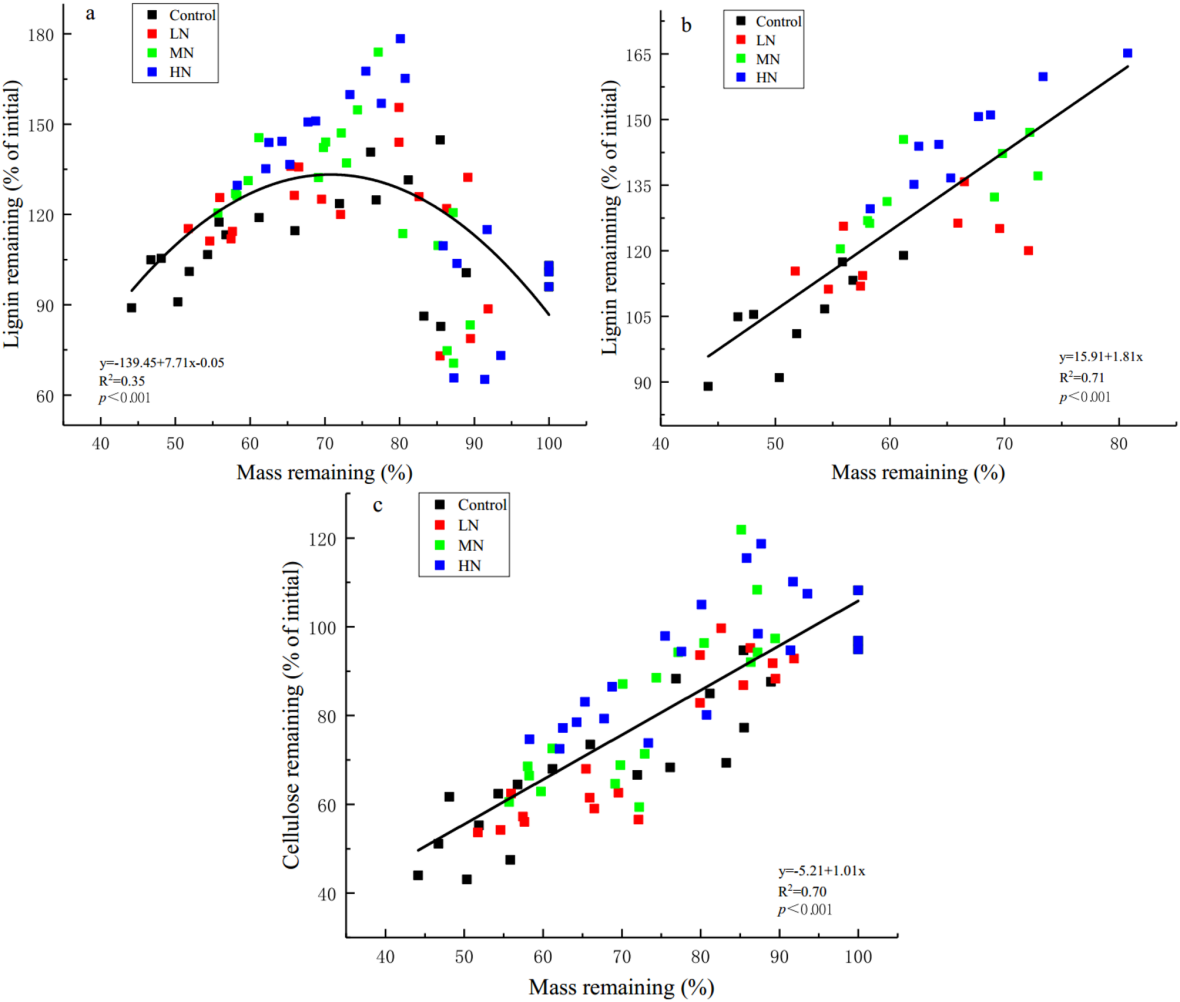
Relationships between the proportion of mass remaining and those of lignin and cellulose remaining. (**a**) Relationship between the proportions of mass remaining and lignin remaining over the experimental period; (**b**) relationship between the proportions of mass remaining and lignin remaining after 6 months of decomposition; (**c**) relationship between the proportions of mass remaining and cellulose remaining over the experimental period. Each point denotes the mean value of three litterbags at each sampling time for each treatment.

The proportion of lignin and cellulose first decreased, then accumulated, and subsequently decreased. The proportion of lignin and cellulose remaining decreased in the initial 2 months; but there was no significant difference in the proportion of lignin remaining between the N and control treatments (Fig. 2a). Lignin and cellulose accumulated, and their respective proportions began to decrease after the 6th and 4th months of decomposition, respectively. After 1 year of decomposition, the proportion of lignin and cellulose remaining significantly increased as N levels increased (Table 1). Linear and nonlinear regression models were used to fit the relationships between the proportion of mass remaining andlignin and cellulose remaining. After 6 months of decomposition, there was a significant positive linear relationship between the proportions of mass remaining and lignin remaining (r^2^ = 0.70, *P* < 0.001; Fig. 3b) and cellulose remaining (throughout the experiment) (r^2^ = 0.71, *P* < 0.001; Fig. 3c) across all N treatments. However, there was no significant positive linear relationship between the proportions of mass remaining and lignin remaining during the experiment (Fig. 3a). Taken together, these results indicate that lignin plays an important role in affecting the loss of leaf litter mass at the later stage of decomposition.

### Effects of simulated N deposition on C/N, C/P, lignin/N, and cellulose/N ratios during leaf decomposition

**Figure 4:**
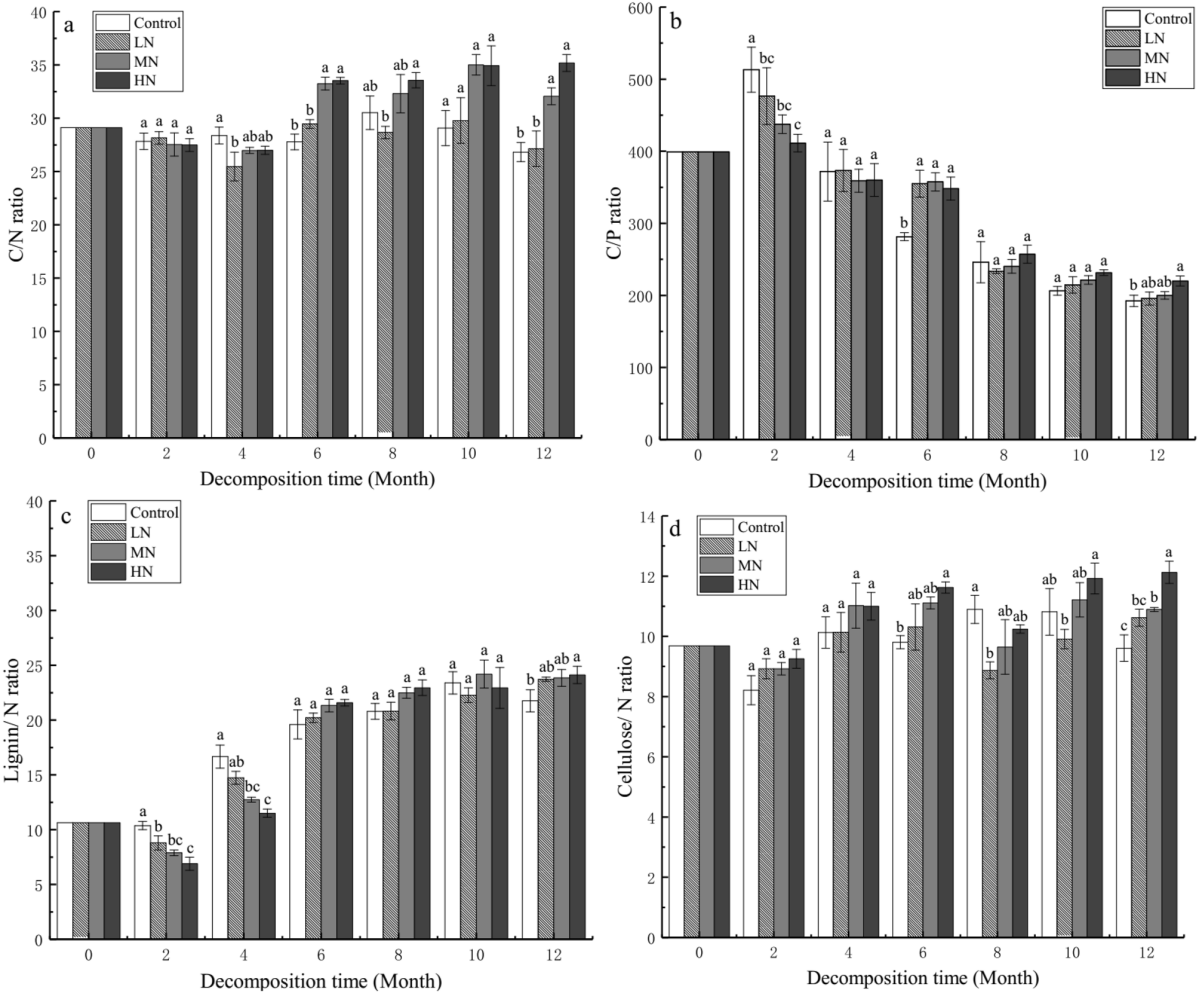
Changes in carbon (C)/nitrogen (N), C/phosphorus, lignin/N, and cellulose/N ratios with different nitrogen treatments during leaf litter decomposition. Values are expressed as mean ± standard error (SE) (n = 3). Values marked with different letters were significant (*P* < 0.05).

The change in C/N ratio between the LN and control treatments was not significant (Fig. 4a). In LN and control treatments, the C/N ratio ranged from 25.45 to 29.78 and 26.82 to 30.51, respectively. After 6 months of leaf litter decomposition, the C/N ratio in MN and HN treatments increased, ranging from 32.06 to 35.01 and 33.52 to 35.17, respectively. C/P ratio decreased during leaf litter decomposition, ranging from 192.55 to 513.09 (Fig. 4b). In the initial 4 months, the lignin/N ratio in the control treatment was significantly higher than that in N treatment (*P* < 0.05). As the decomposition time increased, the lignin/N ratio increased in all treatments, and as the leaf litter decomposition time increased to 12 months, the lignin/N ratio in N treatment was significantly higher than that in control treatment (*P* < 0.05; Fig. 4c). The cellulose/N ratio in each treatment did not significantly differ during the initial 4 months (Fig. 4d); however, after 1 year, it was significantly higher than that in the control treatment (*P* < 0.05).

The results showed that after 1 year of decomposition, the C/N and C/P ratios in the LN, MN, and HN treatments increased by 0.32% (*P* < 0.05), 5.24% (*P* < 0.05), and 8.35% (*P* < 0.05) and 3.13% (*P <* 0.05), 7.45% (*P* < 0.05), and 27.47% (*P* < 0.05), respectively, compared with those in the control treatment (Table 1). Furthermore, the lignin/N and cellulose/N ratios in these treatments increased by 1.97% (*P* < 0.05), 2.08% (*P* < 0.05), and 2.37% (*P* < 0.05) and 1.01% (*P* < 0.05), 1.29% (*P* < 0.05), and 2.51% (*P* < 0.05; Fig. 4c,d), respectively, indicating that N treatments significantly increased the C/N, C/P, lignin/N, and cellulose/N ratios in leaf litter by the end of the experiment.

### Soil properties under N input during leaf litter decomposition

**Figure 5:**
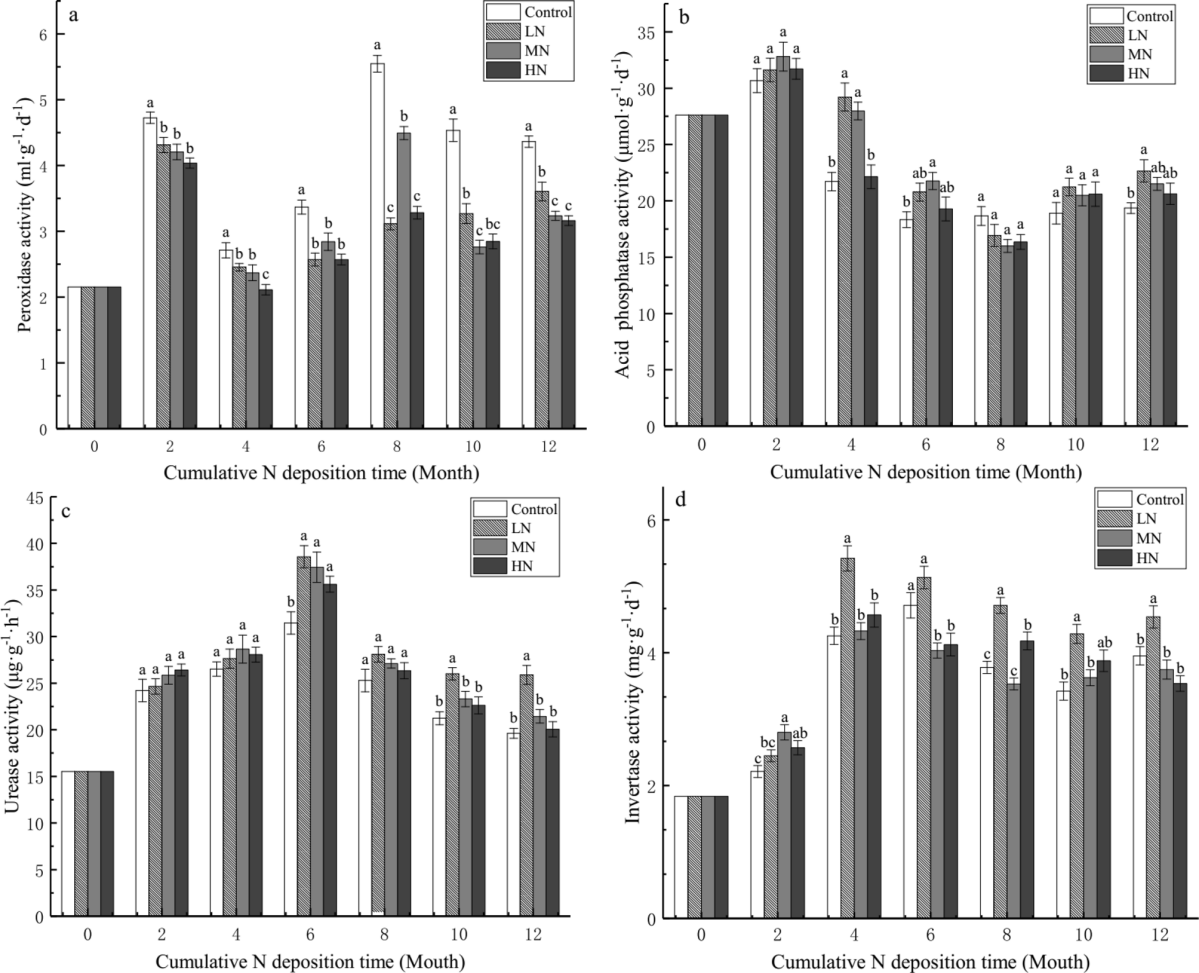
Effect of simulated nitrogen deposition on soil enzyme activity during leaf litter decomposition. Values are expressed as mean ± standard error (SE) (n = 3). Values marked with different letters were significant (*P* < 0.05).

Compared with the initial peroxidase activity, with the input of exogenous N, soil peroxidase activity increased in all treatments (Fig. 5a), and its activity in the control treatment was significantly higher than that in other treatments during the whole experiment (*P* < 0.05). This indicates that N treatments inhibit soil peroxidase activity and that the inhibitory effect increases with increasing N deposition levels. Soil acid phosphatase activity increased during the initial 2 months and gradually decreased thereafter. After 1 year of N input, soil acid phosphatase activity in all experiments was lower than its initial level (Fig. 5b); however, its activity was significantly higher in the N treatments than in control treatment (*P* < 0.05). N treatments increased soil urease and invertase acticities. Urease activity was significantly higher in LN treatments than in control treatment at 6, 10, and 12 months of treatment (*P* < 0.05), with maximum activity during the initial 6 months. Further, soil urease activity was higher in MN and HN treatments than in control treatment (Fig. 5c); however, the difference was not significant except in the 8th month (*P* > 0.05). The highest soil invertase activity was observed in the initial 4 months. Invertase activity was significantly higher in LN treatment than in the other treatments until the end of the study. However, soil urease activity was lower in MN and HN treatments than in control treatment at the end of the study; nevertheless, the difference was not significant (*P* > 0.05).

## Discussion

### Effects of simulated N deposition on leaf litter decomposition and elements release

Reports on the effect of N deposition on leaf litter decomposition are conflicting; generally, this effect is mutually affected by litter quality and exogenous N supply^17^. Simulated N deposition significantly affected the leaf litter decomposition of *C. migao*. N input suppressed the loss of leaf litter mass (Table 3) and significantly increased the proportion of mass remaining (Table 1, Fig. 1a). Furthermore, after 1 year of decomposition, N input significantly reduced the *k*-value. The T_50%_ and T_95%_ of the leaf litter of *C*. *migao* after LN, MN, and HN treatments increased compared with those after control treatment (Table 2), and these inhibitory effects increased with increasing levels of N deposition. The effects of different N treatments on the proportion of mass remaining mainly depended on the decomposition time, and the inhibitory effect of N deposition on the proportion of mass remaining usually increases with time^17^. We also found that time significantly affected leaf litter decomposition (Table 3). The negative effects of N input on leaf litter decomposition were consistent with those reported by Feng et al.^45^ and Diepen et al.^46^, verifying hypothesis 1 of our experiment. In addition, *C*. *migao* leaf contains many phenolic compounds^44^, and a previous study has shown that microbial decomposers could easily form resistant compounds with exogenous N when degrading polyphenolic compounds during litter decomposition^47^. Therefore, we inferred that this might be one of the major drivers to governing the inhibitory effect of N deposition on litter decomposition of *C*. *migao*; however, further research is needed to confirm this.

**Table 3:** Results (F-value) of repeated measures ANOVA of the effects of N deposition, time, and their interactions on mass remaining, elements remaining, organic material remaining, C/N and C/P ratios, and soil enzyme activity during leaf litter decomposition.

Source	Mass remaining	Element remaining (%)			Organic remaining (%)		C/N	C/P	Soil enzyme activities			
		Carbon	Nitrogen	Phosphorus	Lignin	Cellulose			Peroxidase	S-ACP	Urease	Invertase
N	52.02**	103.37**	85.58**	61.82**	87.46**	94.43**	6.78	0.061	136.49**	52.85*	101.20**	228.17**
T	302.83***	580.19***	280.17***	12.53*	75.51*	75.56**	19.26*	51.71**	528.77***	338.45***	468.70***	479.36***
N × T	2.22	16.58*	1.90	4.33	48.44*	3.26	3.81	2.20	23.57*	15.93*	11.92*	69.60**

*N* nitrogen deposition; *T* decomposition time (month); *N* × *T* interactions of nitrogen and time. Values of probabilities (*P*) for repeated measures ANOVA in bold are significant (*P* < 0.05. *, *P* < 0.05; **, *P* < 0.01, ***, *P* < 0.001). S-ACP: Acid phosphatase.

Litter quality is one of the main factors regulating litter decomposition; therefore, simulated N deposition may alter the chemical properties of leaf litter^48^, thereby affecting litter decomposition (e.g., decreasing the C/N and lignin/N ratios to indirectly affect litter decomposition)^18,46^. In our study, we observed that the N treatments significantly suppressed the net release of C and N (Table 1, Fig. 1b,c) and increased the proportion of residual P (Fig. 1d, Table 1). The C in litter is mainly in the form of lignin, cellulose, and hemicellulose. The inhibition of lignin and cellulose decomposition can promote "carbon sequestration," thereby increasing the C content in litter^49^. N treatments significantly increased residual C of *C. migao* litter; this result could be explained by the inhibition of lignin and cellulose at the later stage of decomposition, consistent with the findings of previous studies^28,50^. Litter decomposition is primarily regulated by C/N ratio^51^. Previous studies have reported that irrespective of their initial values, the critical C/N ratio in broad-leaved trees is 20–30 and that N immobilization usually occurs beyond this threshold^52,53^. Therefore, C/N ratio can be used as an important indicator of whether N is released or immobilized during litter incubation^37^. In the present study, we observed that the C/N ratio in the MN and HN treatments started increasing after 6 months of decomposition and continued until the end of the experiment. The highest C/N ratios were around 35 (Fig. 4a, Table 1), indicating that chemical N immobilization, and/or microbial N fixation occur in leaf litter at the later stage of the experiment^54^. This was similar to the results of a study on *C*. *camphora* litter decomposition^37^. Moreover, the higher the C/P ratio in the litter, the lesser P released during decomposition, reducing the microbial biomass^17^. According to the theory of ecological chemometrics, microbial growth is usually limited when the C/P ratio is > 186^55^; in our study, C/P ratio was higher than this threshold during the experiment (Fig. 4b). We infer that N treatment may inhibits the activity of microbial decomposers related to P decomposition and increases the limitation of P release during litter decomposition^17^. Therefore, the release pattern of elements from *C*. *migao* leaf litter indicates that simulated N deposition treatments alter leaf elemental stoichiometry to affect the decomposition of leaf litter, thereby supporting our second hypothesis.

### Effects of simulated N deposition on lignin and cellulose of leaf litter decomposition

As decomposition proceeds, the cellulose level rapidly decreases, whereas lignin often accumulates in litter over time^31^. Excessive proportions of N may affect litter decomposition, particularly by slowing down the decomposition of complex biochemical substances, such as lignin^56^. Consistent with previous findings^28^, we found that simulated N deposition significantly inhibited lignin and cellulose decomposition (Fig. 2a,b; Table 1). Litter decomposition is mainly mediated by extracellular microbial enzymes that directly break down litter cellulose and lignin^17^. N input affects microbial biomass, microbial communities, and the subsequent release of specific extracellular enzymes (e.g., a shift from a highly efficient fungus-dominated community to a less efficient bacteria-dominated community)^28,57^, as well as the synthesis of lignin-degrading enzymes by fungi (particularly the white-rot fungi). This indirectly affects the quality and decomposition of litter^58^. A study on *C. camphora* litter decomposition indicated that the most abundant endophytic fungi in leaves significantly influence leaf decomposition^59^. Jiang et al. also found that the relative abundance of *Ascomycota* and other fungi plays an important role in the decomposition of *Pinus tabulaeformis* litter^33^. Conversely, periodic N input has a negative impact on the decomposition of lignin and modified lignin-like humic products, resulting in a substantial amount of residues^31^. Cellulose decomposition under N treatments was inhibited in the later stage of decomposition, possibly because a part of cellulose was surrounded by lignin and protected by lignin polymers; therefore, the slow decomposition of lignin also suppresses cellulose decomposition^60^. Lignin decomposition was traditionally thought to increase during mid-stage litter decomposition, when cellulose occlusion by lignin began to limit mass loss^61^. Furthermore, after 6 months of decomposition, there was a significant positive linear relationship between the proportion of lignin remaining (Fig. 3b) and cellulose and mass remaining (Fig. 3c); this suggests that lignin forms the main constituent of the residual litter during decomposition, this is consistent with the findings of previous studies^28,62^. Moreover, the litter decomposition rate generally negatively correlates with the lignin/N ratio^56^. The results of in situ litter decomposition experiments by Wang et al. suggest that N input decreases the lignin/N ratio to increases litter decomposition^43^; however, we found that N input significantly increased lignin/N ratio compared with that in control treatment at the later stage of decomposition (Fig. 4c), indicating that N input may change the lignin/N ratio to decrease the decomposition rate of the leaf litter of *C*. *migao*^56^. Chronic N enrichment can decrease the concentrations of other nutrients, such as Mn and Ca, in leaves, limiting the production of ligninolytic enzymes; this affects litter decomposition^46^. Therefore, our next N treatment study should focus on the changes in other nutrient levels, enzyme activities, and their effects on leaf litter decomposition in *C*. *migao*.

### Effects of simulated N deposition on soil enzyme activity during litter decomposition

N deposition can change the structure and function of microorganisms in soil ecosystems, and changes in the microbial community can alter the potential of enzymes to affect litter decomposition and organic matter mineralization^38^. Meanwhile, N deposition in different ecosystems also strongly affects the activities of the enzymes involved in C, N, and P transformation^63^. N input may increase litter decomposition by stimulating microbial activity when soil N availability is low but may inhibit it when soil N availability is high^64^. Continuous excessive N addition can accelerate the loss of NO_3_^−^, loss result in soil acidification as well as increase the solubility of some toxic ions (such as Al^3+^), limiting the metabolic activities in microorganisms. A high N level can inhibit the activities of enzymes related to lignin degradation^65^. For instance, Wang et al. suggested that peroxidase activity positively correlates with an proportion of Trichocomaceae and Chaetomiaceae^38^ because N input may change fungal proportions and peroxidase activity to affect litter decomposition rates^38,66^. Freedman et al. reported that simulated N deposition shifts the saprotrophic microbial community toward bacterial metabolisms that are less oxidatively powerful in lignin decomposition than fungal pathways^67^. The results of the present study are similar; therefore, we inferred that N addition might limit the metabolic activities of peroxidase-related microorganisms (particularly white-rot fungi), which is one of the main reasons for the decrease of peroxidase activity^56^. This may increase the difficulty of lignin degradation in the later stage of degradation. Generally, N deposition can increase microbial activity and soil microbial biomass C in N-limited soil to increase the activities of enzymes related to P^68^. However, N deposition can also decrease acid phosphatase activity to inhibit P mineralization by changing the production and distribution of microorganisms^69^. In the present study, acid phosphatase activity was higher in all N treatments than in control treatment at all points, except in the 8th month of decomposition (Fig. 5b). This result indicates that N treatments limit the release of P from leaf litter to the soil increase the P demand of plants and soil microbes as well as promote the soil P cycle by increasing soil phosphatase activity, regulating P release from the organic matter^68^. N addition can promote soil nitrogen accumulation, and increase the N use efficiency of soil decomposers, thereby improving urease activity^54,70^. Our results showed that N treatment increased the activity of soil urease compared with that in control treatment (Fig. 4c); N input could increase ammonium nitrogen availability in the soil, reduce the decomposition rate of urea by urease, and reduce the number of fungi and actinomycetes^71^. However, excessive N input may have negative effects on soil microbial activities^65^. This could be explained why MN and HN treatments decreased urease acticity compared with LN treatment at the later stage of decomposition. Invertase is an important indicator of soil C cycling rate. N input can change the allocation of microbial resources from nutrient acquisition to C acquisition, which increases the activity of enzymes related to C cycle^43^. In our study, we also found that the invertase activity remained high during the process of litter decomposition under LN treatment. However, the response of soil enzyme activities to N addition was not consistent. Although N treatment increased the activities of acid phosphatase, urease and invertase, and in most cases, there has no significant difference in the activities of acid phosphatase and urease between different treatments and control. Further, the changes in enzyme activity were irregular. This may be due to the limited effects of short-term N addition on soil nutrient balance and soil properties, Frey et al. reported that microbial community and soil physicochemical properties jointly determine substrate utilization patterns and soil enzyme activity^72^. In addition, the irregular changes of soil enzyme activities may be affected by climatic differences; this could be the reason why most enzyme activities exhibited seasonal patterns, suggesting that there is a strong relationship between the degree of microbial activity and the succession and cold, warm, moist, and dry periods in the typical subtropical monsoon climate^37^. In addition, we found that the effects of N input on soil enzymatic activities were dependent on the dominant enzyme species and sampling time (Fig. 5; Table 3), consistent with the results of a previous study^54^.

The effect of N treatment on soil enzyme activity was different^18,71^. Compared with the control treatment, we found that after 1 year of decomposition, the suppression of soil peroxidase activity by N treatment might inhibit lignin decomposition, indirectly affecting litter decomposition. Therefore, our third hypothesis was not fully verified. The effects of N deposition on litter decomposition might be related to other factors, such as the quantity and type of N, forest type, climatic differences, soil fertility and soil layer^18,73^. Therefore, long-term monitoring is required to better understand the changes in soil enzyme activity during leaf litter decomposition of *C*. *migao* with N deposition, the relationship between litter decomposition and soil nutrient cycle, and the relationship between soil enzyme activity and changes in microbial community after litter decomposition.

In conclusion, we observed that compared with control treatment, simulated N deposition distinctly changed the chemical properties and suppressed the decomposition of organic compounds, such as lignin and cellulose. These factors might have inhibited the leaf litter decomposition in *C*. *migao*. Future continuous N deposition may affect the elements cycling of C, N, and P in *C*. *migao*. N input significantly suppressed soil peroxidase activity, which could indirectly affect lignin decomposition in leaf litter. The differences in soil enzyme activities were primarily influenced by enzyme type and sampling time. This may be related to the effects of N input on the availability of soil nutrients, changes in soil microorganisms, and seasonal changes in subtropical areas. Additionally, N deposition might alter soil properties and the abundance and composition of microbial communities, thereby affecting enzyme activity. Therefore, the effect of N input on soil properties, microbial communities, and the activity of soil enzymes on the leaf litter decomposition of *C*. *migao* require further study.

## Material and methods

### Study area

Leaf litter decomposition experiments on *C*. *migao* were conducted in the town of Luokun in Luodian County, Guizhou Province, Southwest China (106°35′ E, 25°17′ N, 735 m a.s.l.). This area is located in the slope zone between the Yunnan–Guizhou Plateau and a hilly area. This topographic environment creates special climatic conditions that form a “natural greenhouse.” Further, it has a subtropical monsoon climate. The average annual temperature is 20.3 °C, maximum monthly average temperature is 32.8 °C, lowest monthly average temperature is 9.2 °C, and average annual rainfall is 1200 mm. The unique geographical location provides an environment conducive for the growth of *C. migao*. Vegetation in this area includes *Photinia parvifolia*, *Vernicia fordii*, *Ligustrum lucidum*, *Alangium chinense*, *Tripterygium wilfordii*, *Rhus chinensis*, *Nephrolepis auriculata*, *Setaria viridis*, and *Ageratina adenophora*.

### Leaf litter sampling and preparation

Leaf litter of *C*. *migao* was collected in October 2017. All samples were air dried at room temperature in the laboratory for 2 weeks until they reached a constant weight. Five samples were randomly selected and oven dried at 70 °C for more than 48 h to determine the initial dry mass and C, N, P, lignin, and cellulose contents, as described later. Before N input, three soil samples were collected from a soil depth of 0–10 cm in each plot to analyze of the initial soil enzyme activity. After oven drying, 10 g of uniformly mixed samples of leaf litter was randomly weighed and placed into nylon mesh decomposition bags sized 20 cm × 15 cm and with upper and lower surface apertures of 0.05 mm. Three sample bags were used for each replicate.

### Leaf litter decomposition experiment and sampling

N input treatments were initiated in 12 plots sized 5 m × 4 m in January 2018, with a 2-m buffer zone between sample plots to prevent interference. There were 648 litterbags in total (4 N levels × 6 sampling times × 9 bags per sampling time) and 54 bags per plot. Before using the decomposition bag, the litter layer on the surface was removed, and the nylon bag was laid on the ground to decompose the sample as closely as possible to its natural state. According to the N deposition in Southwest China (< 15 g·m^−2^·year^−1^ in Guizhou), four N treatments were set up with three replicate plots per treatment: control (CK: 0 g·m^−2^·year^−1^), low N (LN: 5 g·m^−2^·year^−1^), medium N (MN: 15 g·m^−2^·year^−1^), and high N (HN: 30 g·m^−2^·year^−1^). NH_4_NO_3_ needed for each plot was dissolved in 6 L of water and sprayed onto the LN, MN, and HN plots evenly using a spraying device^74^. The same amount of water was sprayed onto the control plots. N fertilization was first applied in January 2018 and the same amount was applied in March, May, July, September, and November 2018. Samples were collected every 2 months after installation. Nine bags (inclusive of three replicates) were randomly selected from each treatment, and soil and debris were removed from the surface of the nylon bags. After oven drying at 65 °C to a constant weight, the chemical contents of leaf litter were determined. Meanwhile, soil samples 0–10-cm deep were randomly collected from the soil center where the decomposition bags were located in each sample plot. Each treatment was repeated thrice. After sampling using the quartile method, soil was sifted through a 2-mm sieve, sealed in self-sealing bags, and stored in a refrigerator at 4 °C for determination of soil enzyme activity.

### Leaf litter chemical composition and soil enzyme activity

Total C content in leaf litter was determined by via potassium dichromate oxidation titration with a Fe^2+^ solution^75^. Total N content was determined by via acid digestion using the Kjeldahl method^76^. Total P content was determined using the molybdenum–antimony colorimetric method after the samples were digested with H_2_SO_4_^77^. Further, lignin and cellulose contents were determined using the acid detergent fiber method with minor modifications^78^. The activities of soil enzymes, including peroxidase, acid phosphatase, urease, and invertase, were estimated using soil enzyme activity kit (Beijing Solebo Biotechnology Co., Ltd.) in accordance with the manufacturer’s instructions. The initial chemical composition of leaf litter and enzyme activity in 0–10-cm soil are detailed in Table S1.

### Data and statistical analysis

The percentage of remaining (*R*) mass, lignin, cellulose, and elements (C, N, and P) in leaf litter during each period (*X*_*i*_) was determined and compared with the initial values (*X*_0_) using the formula *%R* = (*X*_*i*_ / *X*_0_) × 100. The leaf litter decomposition rate constant (*k*) was determined using the Olson’s exponential decay model, i.e., *Y* = *a* × *e*^*−kt*79^, where *Y* is the fraction of remaining mass at time *t* (year^−1^), *a* is the correction factor, *k* is the decomposition rate constant (year^−1^), and *t* is the time (years). The time (years) required for 50% (T_50%_) and 95% (T_95%_) leaf litter decomposition based on Bockheim’s method was calculated as 0.693/ − *k* and 3/ − *k*, respectively^80^.

Homogeneity of variance was determined before performing one-way analysis of variance (ANOVA), and data were logarithmically transformed when required. To assess differences among leaf litter treatments, ANOVA with the least significant difference test was performed to quantify remaining mass, elements (C, N, and P), lignin, and cellulose. Samples were collected every 2 months, and repeated measures ANOVA (N input as the main effect and time as the within-subject factor) was performed to test the remaining mass, remaining elements (C, N, and P), remaining lignin, remaining cellulose, C/N ratio, C/P ratio, and soil enzyme activity to determine the effects of N input, time, and their interactions during litter decomposition. Mauchly’s test of sphericity was performed to validate whether the data conformed to the equal variances for repeated measures ANOVA, and if they were not fulfilled, data were adjusted using the Greenhouse–Geisser method. Linear and nonlinear regression models were used to fit the relationships between remaining mass and remaining lignin and cellulose during litter decomposition. Statistically significant differences were set at α = 0.05, and all parameters were analyzed using SPSS version 21.0 statistical package (Chicago, IL, USA). All presented data are shown as means and standard errors of at least three replicates. Graphs were constructed using Origin 9.0 (Origin Lab, Northampton, MA, USA).

## Supplementary Information

Supplementary Information 1.

## Abbreviations

C: Carbon
N: Nitrogen
P: Phosphorus
LN: Simulated low nitrogen deposition
MN: Simulated medium nitrogen deposition
HN: Simulated high nitrogen deposition
