# Supplementary material for: Simulated atmospheric nitrogen deposition inhibited the leaf litter decomposition of Cinnamomum migao H. W. Li in Southwest China
Source: Sci Rep. 2021 Jan 18;11:1748. doi: 10.1038/s41598-021-81458-3 (PMC7814063; doi:10.1038/s41598-021-81458-3)
Supplement: Supplementary file 1 — Supplementary Information 1. [file 41598_2021_81458_MOESM1_ESM.docx]

**Simulated atmospheric nitrogen deposition inhibited the leaf litter decomposition of  *Cinnamomum migao* H. W. Li in Southwest China**

Xiao-Long Huang,^1,2,†^ Jing-Zhong Chen,^1,2,†^ Deng Wang,^1,2^ Ming-Ming Deng,^1,2^ Meng-Yao Wu, ^1,2^ Bing-Li Tong, ^1,2^ Ji-Ming Liu ^1,2,✉^

^1^ Department of Ecology, College of Forestry, Guizhou University, Guiyang 550025, China

^2^ Forest Ecology Research Center of Guizhou University, Guiyang 550025, China

✉Corresponding author: Department of Ecology, College of Forestry, Guizhou University, Guiyang

e-mail: [karst0623@163.com](mailto:karst0623@163.com); Tel.: +86 13985015398

^†^ Equal contribution to the study

**Table S1** Initial chemical composition of the leaf litter and enzyme activity in the tested soil samples (0–10 cm) (mean ± SE, n = 3)

| Total C  (g·kg^−1^) | Total N  (g·kg^−1^) | Total P  (g·kg^−1^) | Lignin  (g·kg^−1^) | Cellulose  (g·kg^−1^) | pH | Peroxidase  ml·g^−1^·d^−1^ | Urease  μg·g^−1^·h^−1^ | Phosphatase  μmol·g^−1^·d^−1^ | Invertase  mg·g^−1^·d^−1^ |
| --- | --- | --- | --- | --- | --- | --- | --- | --- | --- |
| 594.90 ± 7.75 | 20.43 ± 0.19 | 1.51 ± 0.12 | 217.23 ± 4.55 | 197.83 ± 8.20 | 5.09 ± 0.07 | 2.15 ± 0.21 | 15.53 ± 0.39 | 27.62 ± 0.57 | 1.84 ± 0.04 |
